# Bedtime Salivary Cortisol and Depressive Symptoms in Older Adults Living With Dementia

**DOI:** 10.1093/geroni/igab046.1691

**Published:** 2021-12-17

**Authors:** Fanghong Dong, Miranda McPhillips, Darina Petrovsky, Liming Huang, Adriana Adriana, Nancy Hodgson

**Affiliations:** 1 UPenn, Philadelphia, Pennsylvania, United States; 2 University of Pennsylvania, University of Pennsylvania, Pennsylvania, United States; 3 Rutgers University, Philadelphia, Pennsylvania, United States; 4 University of Pennsylvania, Philadelphia, Pennsylvania, United States; 5 University of Pennsylvania, School of Nursing, philadelphia, Pennsylvania, United States

## Abstract

The dysregulation of cortisol has been associated with depressive symptoms in older adults. To date, no prospective longitudinal studies have examined whether salivary cortisol is a risk factor for depressive symptoms in persons living with dementia (PLWD). With a sample of 123 PLWD, baseline salivary cortisol was collected at awaking, 30 minutes after awaking, and bedtime. Depressive symptoms were assessed at baseline and the four-week follow-up. Cortisol indicator were centered. Baseline bedtime cortisol level was significantly associated with depressive symptoms in a curvature style while controlling age, gender, and baseline depressive symptoms (β=3.76 for linear term and β=-1.57 for quadratic term, both ps<0.04). No other baseline cortisol measures were significant prospective predictors. Our results suggest the bedtime cortisol was a significant risk factor for depressive symptoms in PLWD. These findings suggest that bedtime cortisol may play a role in the etiology of depressive symptoms in PLWD.

